# Implementing an Interprofessional Simulation Program for Paediatric Patient Safety

**DOI:** 10.1111/tct.70301

**Published:** 2026-01-07

**Authors:** David D'Arienzo, Mylene Dandavino, Andrée‐Anne Matte, Stephanie Welsh, Catherine Rich, Nadine Korah

**Affiliations:** ^1^ Department of Pediatrics, Montreal Children's Hospital McGill University Montreal Canada; ^2^ Institute of Health Policy, Management and Evaluation University of Toronto Toronto Canada; ^3^ Institute for Health Professions Education McGill University Montreal Canada

**Keywords:** adverse events, education, errors, interprofessional, multidisciplinary, patient safety, simulation

## Abstract

**Background:**

Simulation‐based medical education is a widely recognised tool to improve patient safety culture and outcomes. Many preventable clinic errors are specific to the unit where they occur. We planned and conducted an interprofessional in situ simulation program aligned with unit‐specific errors and report its design, implementation and evaluation.

**Approach:**

This prospective, survey‐based study was conducted at a paediatric tertiary care centre on a medical–surgical inpatient ward. Incident and accident reports were reviewed; the most frequent and/or severe events were identified. Eight in situ simulations were implemented over 8 months. Participants, including residents, medical students, nurses, pharmacists and physicians completed a survey exploring perceptions of the program's ability to improve patient safety.

**Evaluation:**

Incident reports over 12 months were reviewed (*n* = 329). Four most frequent and serious errors were identified: intravenous solutions selection, enteral feeding rate or type selection, medication transcription and IV infiltration. Simulation participation rate was 86%; survey response rate was 65%. We evaluated the program using Kirkpatrick's model of educational training, assessing reaction through self‐reported surveys and results through changes in incident report frequency. Overall, 86% of participants responded positively regarding the program's potential impact to enhance patient safety. This is supported by an increase in incident reporting the year following program implementation and a decrease in each simulation‐targeted medical error, albeit not statistically significant.

**Implications:**

Our experience in creating an in situ simulation program that aligns with unit‐specific needs and addresses implementation challenges may provide valuable insights for other centres seeking enhance patient safety.

## 1 Background

Patient safety remains a critical priority in paediatric hospitals. Nearly 10% of children admitted to a Canadian paediatric hospital experience one or more adverse events, and 45% of these adverse events are believed to be preventable [1]. Errors in communication and teamwork contribute to preventable harm, underscoring the need for targeted interventions to enhance interprofessional collaboration [1, 2]. Simulation‐based training has emerged as an effective strategy to improve clinical skills, reinforce teamwork principles and mitigate patient safety risks in high‐stakes environments [2, 3]. Moreover, simulation‐based analysis of safety events can facilitate the identification of previously unrecognised errors and contribute to the development of novel recommendations following the simulation [3].

*Simulation‐based analysis of safety events can facilitate the identification of previously unrecognised errors*.

Interprofessional simulation activities provide a structured, experiential learning environment where healthcare providers (HCPs) from different disciplines can practice managing clinical scenarios in a safe setting [4]. These exercises facilitate the development of shared mental models, enhance situational awareness and promote effective communication—key factors in reducing medical errors and improving patient outcomes [5, 6]. In situ simulations or those conducted in actual clinical environments rather than in simulation centres offer a valuable opportunity to enhance learning and identify latent safety threats related to equipment malfunctions/inaccessibility, workflow inefficiencies, communication breakdowns or system vulnerabilities [6, 7, 8]. By testing real teams, equipment and workflows within the authentic clinical environment, system issues that may not be apparent in centre‐based settings are identified [9]. By immersing interprofessional teams in realistic patient care scenarios, in situ simulations align with interprofessional education frameworks and help reinforce critical skills while uncovering operational challenges that may compromise patient safety [7, 10, 11, 12]. Despite their advantages, logistical challenges such as scheduling, resource availability and potential disruptions to clinical workflows can make the implementation of in situ simulation programs complex. However, when effectively integrated into hospital systems, in situ simulations provide actionable insights that drive quality improvement initiatives and strengthen institutional safety cultures [13].

We describe the design and implementation of a unit‐specific interprofessional, in situ simulation program that aimed to enhance patient safety in a paediatric tertiary care hospital, informed by the recurrent patient safety incidents of that specific inpatient unit. We discuss how logistical challenges were addressed to maximise the program's impact. We also evaluate participants' perceptions of the program's potential impact as a medical education tool for enhancing patient safety culture. Our simulation program is particularly innovative as it combines interprofessional simulations, a context‐specific medical errors curriculum and in situ practices.

*We describe the design and implementation of a unit‐specific interprofessional, in situ simulation program that aimed to enhance patient safety*.

## 2 Approach

This was a prospective survey‐based study conducted at a Canadian tertiary care paediatric hospital. Simulations were designed following a review of all incident reports submitted over a 12‐month period on medical–surgical paediatric units at a tertiary care paediatric hospital. Simulations were designed based on the four most common errors. These included (i) selection of IV solution 4.0% (*n* = 13), (ii) selection of feeding rate or type 7.6% (*n* = 25), (iii) medication transcription 7.0% (*n* = 23) and (iv) IV infiltration 4.9% (*n* = 16). These errors are similar to the most common errors reported in other inpatient settings [12]. The simulations were designed so that the safety error event was the critical event in each scenario. For example, an 18‐month‐old girl admitted with bronchiolitis started having a tonic–clonic seizure in the context of hypoglycaemia, because she was receiving normal saline as her intravenous fluid for over 24 h and not D5NS (which contains dextrose), which would be standard of care for an infant not tolerating feeds.

Simulation design followed the Healthcare Simulation Instructional Design Framework, which includes sequential phases of **analysis**, **design**, **development**, **implementation** and **evaluation** [14]. Analysis included the review of prior incident reports previously discussed. Simulations were designed and developed by an interprofessional team consisting of nurses, physicians, trainees and pharmacists. Key objectives and content were defined, focusing on the safety error event as the key topic. Simulations were refined through review by subject‐matter experts to ensure clinical fidelity and alignment with local practice. Hospitalists with expertise in simulation design, nurse managers and trainees piloted each simulation to test flow, timing and realism prior to implementation. Simulations were iteratively adapted based on feedback received during the debriefing and evaluation. Simulations were implemented in situ with structured prebriefing and debriefing. Post‐simulation debriefings followed the **Gather–Analyse–Summarise (GAS) framework**, providing a structured approach to facilitate participant reflection, analyse team performance and consolidate key learning points for clinical practice [15]. Simulations were facilitated by trained simulation faculty with formal preparation in simulation‐based education. Facilitator teams were kept consistent across sessions to maintain standardisation and reliability.

Simulation participants included HCPs on the clinical teaching unit (CTU) where the adverse events were reported. These included nurses, nursing assistants, medical students, paediatric residents and fellows and pharmacists. Interprofessional simulations were held in the CTU's procedure room—a room equipped similarly to regular patient rooms. Participants were then invited to complete an anonymous survey, evaluating their experience and perceptions of this new medical education tool and its perceived ability to reduce medical errors and improve patient safety culture. Survey items were generated by the research team based on previously published surveys, including the Hospital Survey on Patient Safety Culture (Agency for Healthcare Research and Quality) to assess patient safety culture and attitudes towards patient safety and the Medical School Graduation Questionnaire (Association of American Medical Colleges) to assess teamwork, communication and effectiveness of interprofessional medical education tools [16, 17].

This study was approved by the Institutional Review Board of the McGill University Health Centre.

## 3 Evaluation

Each simulation was performed twice over an 8‐month period (one simulation per month). Participation rate in the interprofessional, in situ simulations was 86% (*n* = 125). Survey response rate was 65% (*n* = 81). Participants included medical students, nursing staff, residents, fellows and pharmacists (Table 1). The evaluation was guided by Kirkpatrick's model for evaluating training and education, assessing reactions (self‐reported satisfaction and perceptions of the educational tool) and results (system‐level outcomes—changes in frequency of incident reports pre/post‐intervention) [18].

**TABLE 1:** Baseline characteristics of interprofessional in situ patient safety simulation program.

	*N* (%)
Sex	
Male	15 (19%)
Female	53 (65%)
No response	13 (16%)
Age (years)	
18–24	21 (26%)
25–34	48 (59%)
35–44	8 (10%)
45–54	4 (5%)
> 55	0 (0%)
Profession	
Medical resident	25 (31%)
Medical student	19 (23%)
Nurse/nursing assistant/nursing students	36 (44%)
Pharmacist	1 (1%)
Years in practice (years)	
< 1	22 (27%)
1–5	50 (62%)
6–10	4 (5%)
11–20	4 (5%)
21–30	1 (1%)
> 31	0 (0%)

Nearly all participants (98%, *n* = 79) agreed or strongly agreed with all questions pertaining to their perception of the simulation program as a medical education tool that could improve patient safety culture. Additionally, 81% (*n* = 66) of respondents agreed or strongly agreed that the simulation activities would reduce the frequency of medical errors made on the unit. Regarding the simulation program's perceived ability to improve teamwork and communication, 81% (*n* = 66) stated it could positively or strongly positively impact teamwork and interprofessional communication, and 83% (*n* = 67) believed it could positively or strongly positively impact the unit's learning environment.

During the simulations, several latent safety threats were identified. Examples of identified latent safety errors included miscommunications in feeding orders due to orders in multiple locations, which led to discrepancies and errors in order communication and implementation. Uncertainty regarding appropriate IV fluid composition and concentration was also noted due to limited access to reference tools and incomplete emergency medication sheet labelling. Each threat was reviewed with clinical leadership, leading to mitigation strategies such as updating reference materials, implementing double‐check processes for medication transcription, facilitating and streamlining feeding prescriptions, reinforcing escalation pathways for IV complications and integrating these lessons into staff orientation and competency assessments.

*During the simulations, several latent safety threats were identified*.

To evaluate the potential impact of the implementation of the simulation program on the proportion of patient care–related adverse events, the incident reports for the 1‐year period post‐intervention (May 2018 to April 2019) were attained from the hospital's Quality and Safety Office. We collected data on the number of specific error types and the total number of all‐cause events for Year 1 (pre‐intervention, May 2016 to April 2017) and Year 2 (post‐intervention, May 2018 to April 2019). The chi‐square test was used to assess for a difference in the annual proportion of each error type in the year prior to and the year after implementation of the simulation program. A *p* value less than 0.05 was considered statistically significant. Although most specific error types did not see a statistically significant decrease, there was a decrease in the proportion and absolute number of each specific error targeted by the simulation program in the 1‐year period following implementation of the patient safety simulation program, whereas there was an overall increase in the number of total incidents and accidents reported (Table 2). This suggests that teams have learned to identify and address incidents correctly and that more HCPs are reporting patient care issues and medical errors, which can be an indicator of enhanced safety culture as HCPs feel they can speak up [19]. This is further supported by multiple studies that demonstrated that a positive safety culture consistently leads to higher rates of incident and near‐miss reporting among healthcare professionals [20, 21, 22].

**TABLE 2:** Frequency of errors in the 1‐year pre‐intervention versus 1‐year post‐intervention.

	Pre‐intervention (2016–2017)	Post‐intervention (2018–2019)	*p*
IV solution	13	9	0.24
Feeding rate	25	15	0.04*
Medication transcription	23	16	0.11
IV infiltration	16	12	0.026*
*Total reported errors*	***328***	***375***	

* A *p* value < 0.05 was considered statistically significant.

*Teams have learned to identify and address incidents correctly*.

## 4 Implications

This study has important implications for patient safety, interprofessional education and quality improvement across inpatient settings. By identifying the most common errors and integrating them into simulation‐based training, this innovative approach aims to reduce preventable harm on that unit. In situ simulations help teams recognise latent safety threats related to equipment, processes and system inefficiencies, ultimately enhancing overall patient safety. Additionally, interprofessional simulation training strengthens teamwork and communication, fostering a culture of collaboration that improves coordination in high‐risk clinical scenarios [3, 4, 23].

*Identifying the most common errors and integrating them into simulation‐based training, this innovative approach aims to reduce preventable harm*.

Furthermore, by integrating simulation findings into continuous quality improvement efforts, such as morbidity and mortality reviews or patient safety committees, hospitals can implement iterative interventions and sustain a culture of learning and safety. Although the simulation scenarios were developed based on unit‐specific errors and incidents, future simulations could expand to encompass broader aspects of paediatric safety to enhance generalisability and comprehensiveness.

The major challenges associated with conducting in situ, interprofessional simulations and how we addressed each of these issues are highlighted below and summarised in Table 3.

**TABLE 3:** Challenges and proposed solutions to developing an interprofessional, in situ simulation program aimed at improving patient safety.

Challenges	Proposed solutions
1 Time where all healthcare professionals are available	Interprofessional organisation team, who know each professional's schedules, including busy periods (i.e., patient rounds) and breaks
2 Disruption of patient flow and daily activities	Teams consider this simulation as their daily teaching activity. Plan for nursing backup. Calendar invites sent out to team leaders that allow teams to anticipate and protect a specific time for the activity and plan daily activities around the simulation
3 Need to take time away from patients/responsibilities	Total length of time of 30 min allows for: prebrief, simulation and debrief
4 Lack of physical space to run the simulations	Using unused physical space on the ward (i.e., procedure room) while keeping context fidelity
5 Realism of materials and equipment used	Use of low fidelity mannequins and equipment readily available on the units where the simulations took place
6 Managing large groups in short simulations	Used structured facilitation (e.g., GAS debriefing framework) and focused learning objectives to maintain engagement and feasibility; future sessions may use smaller groups for deeper reflection
7 Limitation of self‐reported perceived data	Self‐reported surveys captured reactions only; learning and behaviour can be evaluate through structures observations/validated performance checklists to measure change in knowledge/skills and direct clinical observations/follow up assessments

## 5 Lessons Learned

### 5.1 Challenge 1: No Common Time When All HCPs Are Available

Previous studies have had high rates (28%–67%) of simulation activity cancellations, given the unexpected schedules of different teams, heavy patient loads and shortage of staff [16, 23]. Participation rates of simulations are also extremely variable, often between 20% and 38% [23]. We believe our participation rate was higher due to our interdisciplinary planning team, leadership support at all levels to ensure this activity is protected and mandatory, and a protected time to run this activity. The health profession education and patient safety expertise of our planning team allowed us to anticipate and address logistical issues among the various healthcare teams.

### 5.2 Challenge 2: Disruption of Patient Flow and Daily Activities

The interdisciplinary planning team scheduled the simulations around patient rounds and break times to minimise the effect on routine work‐related activities. Considering the simulation as the protected teaching session and planning for multiple backup nurses from two paediatric medical–surgical inpatient units has decreased the impact on patient flow coming from the emergency department, ICUs and clinics.

### 5.3 Challenge 3: Time Away From Patients/Responsibilities

The short duration (30 min) of the activity allowed them to be conducted easily during the clinical teams' workday. Having support from nursing, pharmacy and medical leadership set a culture valuing simulation and allowed for a scheduled time and place to conduct the program and ensure participation from all groups on a weekly basis, consistently.

*Support from nursing, pharmacy and medical leadership set a culture valuing simulation and allowed for a scheduled time*.

### 5.4 Challenge 4: Lack of Physical Space to Run the Simulations

Using a physical space that was part of the work area made the integration of these activities easy for the clinical teams, due to minimal time loss for displacement, while keeping the context fidelity. Maintaining proximity to the hospitalised patients also allowed members of the medical teams to participate in the sessions.

### 5.5 Challenge 5: Realism of Materials and Equipment Used

Using our procedure room, a space on the inpatient ward where admitted patients go for minor procedures (i.e., lumbar punctures) and which is equipped with the materials routinely found in a patient room allowed us to perform the simulations on the clinical unit without depending on the availability of patient rooms. A low‐fidelity mannequin allowed for easy set‐up and flexible scenario use. Expired equipment and medications would often be used to optimise resource stewardship, and an iPad app called ‘iSimulate’ allowed us to simulate a patient monitor while having the ability to change the vitals throughout the simulated scenario.

### 5.6 Challenge 6: Large Interprofessional Groups in Short Simulations

Managing large interprofessional groups within short simulation sessions posed logistical and coordination challenges, particularly in ensuring active engagement and meaningful debriefing across disciplines. To address this, we used structured debriefing strategies and focused on learning objectives to optimise engagement and maintain feasibility within operational constraints. Although this approach allowed broad participation, future iterations may benefit from smaller groups to allow for deeper reflection and skill consolidation across professions.

### 5.7 Challenge 6: Limitations of Evaluating Perceived Outcomes

Participants' reaction was evaluated through the use of self‐reported surveys, which may be influenced by social desirability bias. Perception‐based measures capture participants' experiences and perceived learning but may not fully reflect objective changes in clinical behaviour or system‐level outcomes. Therefore, the use of self‐reported surveys does not effectively evaluate learning and behaviour. Although these were explored in the debrief, more formal and rigorous assessment tools are needed to evaluate these two levels in Kirpatrick's model.

## 6 Future Directions

Interprofessional in situ simulations, when carefully designed, can help address recurrent patient safety incidents on a clinical paediatric unit and may improve safety event reporting and patient safety culture. By overcoming common challenges to implementing interprofessional in situ simulations, we have expanded the program to include weekly simulations covering over 20 prevalent patient safety incidents. Additionally, the program is now integrated into the training process during the introduction of new clinical protocols on the unit to ensure safe implementation. Our experience in addressing common challenges when implementing interprofessional, in situ simulations may inform other centres looking to implement similar programs aimed at improving patient safety.

*Interprofessional in situ simulations, when carefully designed, can help address recurrent patient safety incidents*.

## Author Contributions

**David D'Arienzo:** conceptualization, data curation, formal analysis, investigation, methodology, project administration, writing – original draft. **Mylene Dandavino:** conceptualization, investigation, methodology, supervision, writing – review and editing. **Andrée‐Anne Matte:** resources, project administration, writing – review and editing. **Stephanie Welsh:** resources, project administration, writing – review and editing. **Catherine Rich:** conceptualization, resources, supervision, writing – review and editing. **Nadine Korah:** conceptualization, investigation, methodology, supervision, writing – review and editing.

## Funding

The authors received no specific funding for this work.

## Conflicts of Interest

The authors declare no conflicts of interest.

## Data Availability

The data that support the findings of this study are available on request from the corresponding author. The data are not publicly available due to privacy or ethical restrictions.
